# LINC00839/miR-144-3p/WTAP (WT1 Associated protein) axis is involved in regulating hepatocellular carcinoma progression

**DOI:** 10.1080/21655979.2021.1990578

**Published:** 2021-11-30

**Authors:** Xingqin Zhou, Yazhou Chang, Lirong Zhu, Chaoyan Shen, Jing Qian, Renan Chang

**Affiliations:** a Department of Radiotherapy, Affiliated Hospital of Nantong University, Nantong, China; b Medical College of Southeast University, Nanjing, China; c Department of Hepatobiliary Surgery, Affiliated Hospital of Nantong University, Nantong, China

**Keywords:** LINC00839, miR-144-3p, WTAP, hepatocellular carcinoma

## Abstract

The present work aimed to explore LINC00839 expression level and its function in hepatocellular carcinoma (HCC), and identify the downstream molecular mechanisms. qRT-PCR (Real-Time Quantitative Reverse Transcription PCR) and western blot were employed to detect mRNA and protein levels. Functional investigations were performed by flow cytometric-based apoptosis assay, CCK8 (Cell Counting Kit-8) assay, clone formation assay, Transwell migration and invasion assay. Functional interactions between LINC00839 and miR-144-3p or miR-144-3p and WTAP were validated by dual luciferase reporter assay. siRNA (small interfering RNA) was used for LINC00839 silencing, and microRNA mimic or inhibitor were employed to modulate miR-144-3p activity. LINC00839 was upregulated in HCC cells and tissues. Silencing LINC00839 suppressed the proliferation, invasion, migration of HCC cells and induced apoptosis. Additionally, LINC00839 served as a sponge to negatively impact on miR-144-3p activity, which contributed to the high expression of WTAP (WT1 Associated Protein) and the malignant phenotype of HCC cells. Our study revealed an oncogenic role of LINC00839 in HCC, and identified miR-144-3p/WTAP axis as downstream effectors mediating the oncogenic function of LINC00839. LINC00839 might serve as a potential therapeutic target and prognostic marker for HCC.

## Introduction:

Hepatocellular carcinoma (HCC) ranks as the third leading cause of tumor-related death and the sixth among newly diagnosed cancer cases worldwide [1]. The pathogenesis of HCC remains unclear, and current treatments are limited by the emergence of drug resistance. Understanding the molecular mechanism of pathogenesis of HCC may facilitate the development of targeted therapy to promote the treatment outcome.

Long noncoding RNAs (lncRNAs) are noncoding RNAs with more than 200 nucleotides, which are implicated in modulating multiple biological processes of malignant tumors [2]. LncRNAs act as competing endogenous RNAs (ceRNAs) to sponge microRNA targets, and attenuate miRNA-mediated regulation of target genes [3]. Previous studies highlighted the dysregulation of lncRNAs in the occurrence and development of HCC, such as TSLNC8, HULC, DANCR, and PRAL [4–7]. LINC00839 was reported to be upregulated in osteosarcoma and breast cancer [8,9]; however, its expression pattern and functional roles in hepatocellular carcinoma remain to be elucidated.

MicroRNAs are a class of non-coding RNAs regulating target gene expression by mediating mRNA degradation or suppressing translation. MiR-144-3p was identified as a novel tumor suppressor in glioblastoma, prostate cancer, cervical cancer, Wilms’ tumor, multiple myeloma, pancreatic cancer, as well as HCC [10–16]. In HCC, microRNA-144-3p acts as a tumor suppressor to suppresses tumor growth and angiogenesis by targeting SGK3 (Serum/Glucocorticoid Regulated Kinase Family Member 3) [17].

RNA is the critical link in the transmission of genetic information from DNA to protein. Different posttranscriptional modifications in various RNAs (including rRNA, tRNA, snRNA, mRNA, and lncRNA) have been characterized, among which N6-methyladenosine (m6A) is the best-studied example [18]. The regulation of RNA m6A modification involves a series of proteins such as writers (methyltransferases), eraser (demethylases), and readers. m6A modification plays a crucial regulatory role in various aspects of RNA metabolism, including stability, positioning, splicing, and translation [19]. Aberrant modification of m6A is implicated in multiple disorders, such as liver cancer [20]. WT1 Associated Protein (WTAP) has been identified as one of the writers (methyltransferases) of m6A modification [21], which facilitates HCC development through m6A-HuR dependent ETS19 silencing [22]. However, the roles of WTAP in regulating HCC phenotype and the association of WTAP with miR-144-3p have been investigated.

In this study, we investigated LINC00839 expression level, and uncovered its functional roles and downstream targets in HCC. We showed that LINC00839 was highly expressed in HCC, and high LINC00839 expression was correlated with a poor prognosis in HCC patients. Through different functional assays, we demonstrated the oncogenic role of LINC00839 in HCC cells. Mechanistically, LINC00839 served as the sponge of miR-144-3p, which relived the suppression on WTAP expression in HCC. Collectively, our data suggest that LINC00839 might serve as a potential therapeutic target and prognostic marker for HCC.

## Material and methods:

### Cell lines and tissue specimens

We obtained HCC cell lines (Huh6, Huh7, SK-hep1, HepG2, and PLC5) and normal hepatocyte cell line LO2 from Shanghai Institutes of Biological Sciences, Chinese Academy of Sciences (Shanghai, China). The cells were cultured in DMEM (Thermo Fisher Scientific, Waltham, MA) containing 10% fetal bovine serum (FBS; Thermo Fisher Scientific) in a humidified incubator under 37°C with 5% CO_2_. Sixty paired HCC samples and matched non-carcinoma tissue samples were obtained in 60 HCC patients by radical resection at the Affiliated Hospital of Nantong University. The Ethics Committee of Affiliated Hospital of Nantong University approved sample collection and the study. Every patient enrolled in this study signed the written informed consent. After collection, liquid nitrogen was used to freeze samples until RNA extraction.

### Bioinformatics prediction using online resources

StarBase v3.0 (http://starbase.sysu.edu.cn/) was utilized for comparing gene expressions in 374 HCC cancer and 50 normal samples (see sFig 1, sFig 2 and sFig 3). DINAN approaches (http://carolina.imis.athena-innovation.gr/diana_tools/web/index.php) and miRcode (http://mircode.org) were employed for predicting LINC00839 targets. The TargetScan online resource (http://www.targetscan.org/vert_71/) was used to identify the potential mRNA targets of miR-144-3p. The survival analysis including over survival (OS) and disease-free survival (DFS) was conducted using the website Kaplan-Meier Plotter (https://kmplot.com/analysis/) using the data of 371 HCC patients.

**Figure 1.**
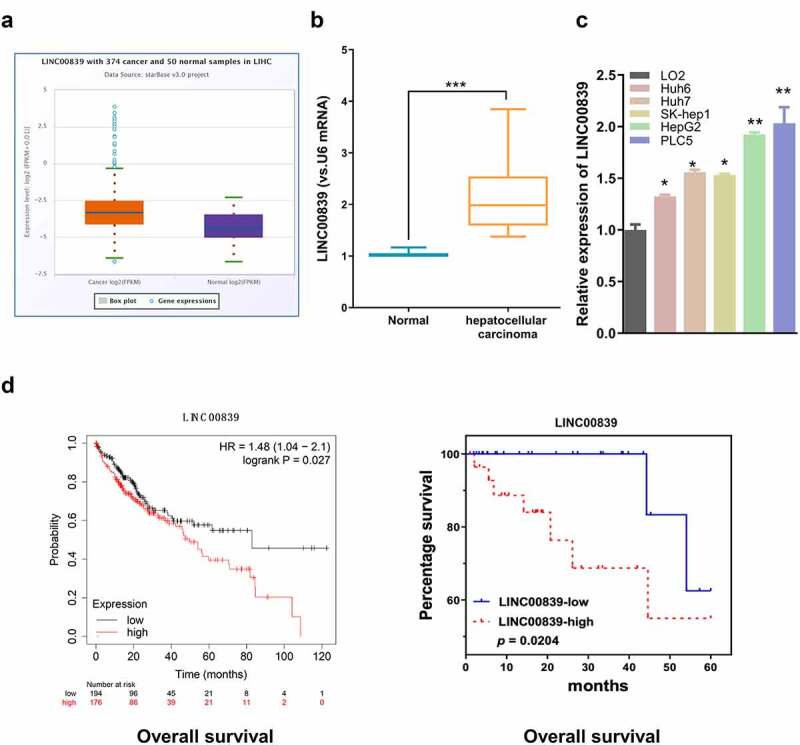
Expression of LINC00839 and its association with HCC prognosis. a) Bioinformatics analysis showed LINC00839 was upregulated in HCC tumor samples from StarBase v3.0. b) High expression level of LINC00839 was confirmed by qRT-PCR in 60 HCC samples and the adjacent normal tissues. c) Expression of LINC00839 was examined in different HCC cell lines (Huh6, Huh7, SK-hep1, HepG2, and PLC5) and normal hepatocyte cell line (LO2). d) High expression of LINC00839 was associated with a poor overall survival according to Kaplan-Meier Plotter with 371 HCC patients (left) and our database with 60 HCC patients (right). The data were shown as the mean ± SD of three independent experiments. *, P < 0.05; **, P < 0.01; ***, P < 0.001

**Figure 2.**
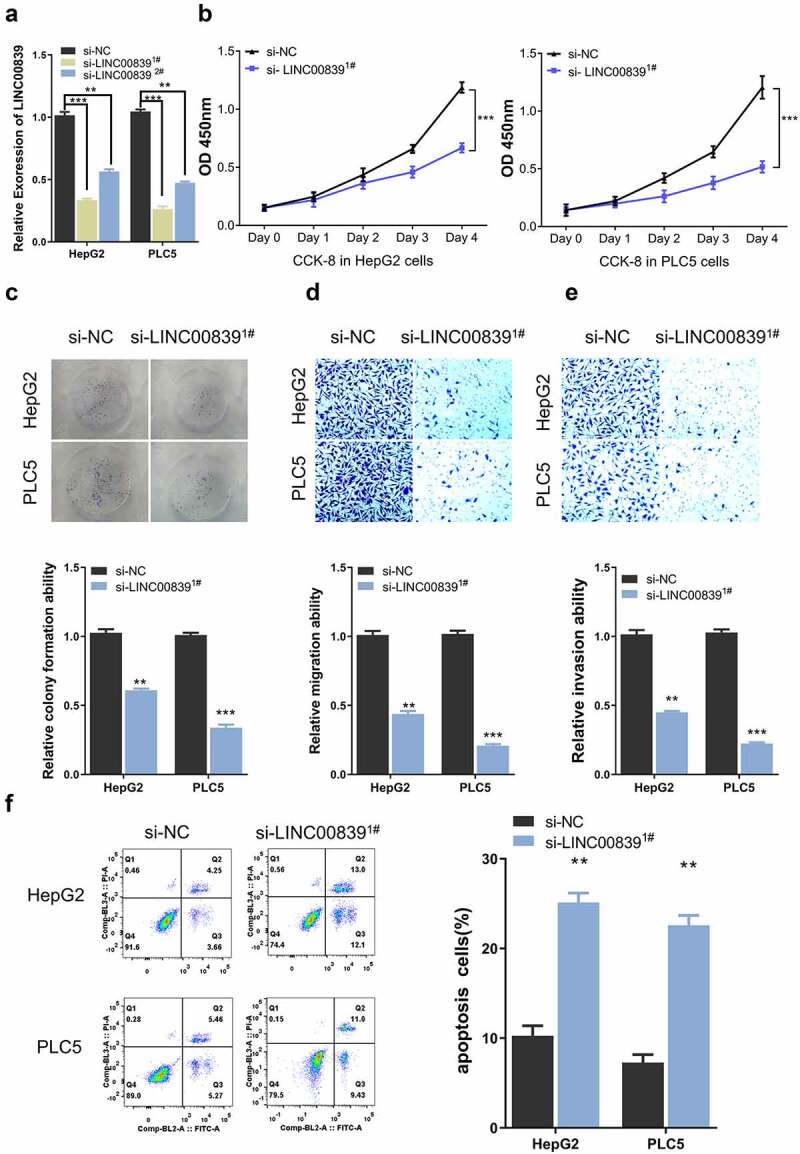
The effect of LINC00839 silencing on cell phenotypes. a) The knockdown efficiency of si-LINC00839^1#^ and si-LINC00839^2#^ by qRT-PCR. b) LINC00839 silencing inhibited cell proliferation in both HepG2 and PLC5 cells. c) LINC00839 silencing inhibited cell colony formation. d-e) Si-LINC00839 impaired cell migration and invasion ability. f) Si-LINC00839 transfection promoted cell apoptosis. The data were shown as the mean ± SD of three independent experiments. **, P < 0.01; ***, P < 0.001

**Figure 3.**
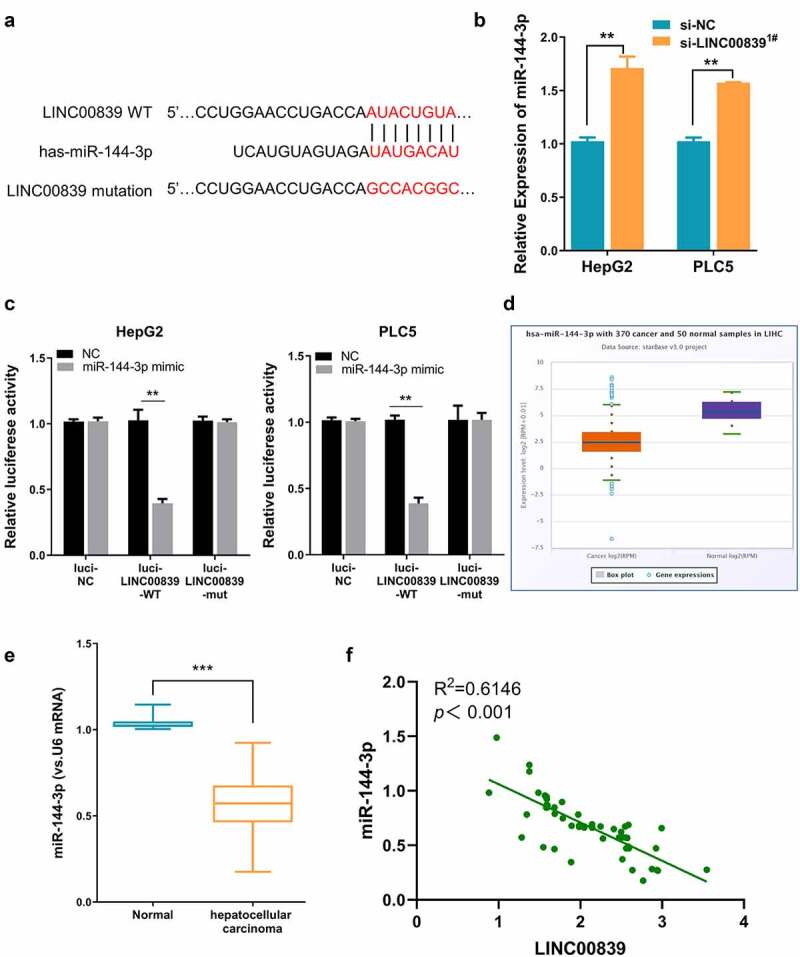
LINC00839 serves as miR-144-3p sponge. a) The predicted binding site of LINC003839 and miR-141-3p. b) LINC00839 silencing upregulated the expression of miR-144-3p in HCC cells. c) Dual-luciferase assay using reporter with wild-type binding site (Luci-LINC00839-WT), mutated reporter (Luci-LINC00839-mut) or empty reporter (Luci-NC) in the presence of MiR-144-3p mimic or miR-NC. d) MiR-144-3p was downregulated in HCC tissues from the database. e) The expression of miR-144-3p was quantified by qRT-PCR in 60 HCC samples and the adjacent normal tissues. f) There was a negative correlation between LINC00839 and miR-144-3p expression in 60 HCC samples. The data were shown as mean ± SD of three independent experiments. **, P < 0.01; ***, P < 0.001

### Vector construction and transfection

LINC00839-targeting siRNA (si- LINC00839) and the scramble control siRNA (si-NC), miR-144-3p inhibitor, mimic as well as negative control (miR-NC) were synthesized by RiboBio Co., Ltd (Guangzhou, China). To construct WTAP overexpression plasmid, the full-length WTAP cDNA was cloned into pcDNA 3.1 vector (pc-WTAP).

Cell transfection was performed using Lipofectamine® 3000 reagent (Thermo Fisher Scientific, CA, USA). In 6 well plates, 70% confluent cells were transfected with 100 nM of microRNA mimic or inhibitor, or 6 ug of pcDNA3.1-WTAP plasmid according to manufacturer’s instruction. Transfected cells were subjected to subsequent experiments 48 hours post-transfection.

### Quantitative Reverse Transcription–Polymerase Chain Reaction (qRT-PCR)

Trizol reagent (Thermo Fisher Scientific) was used to extract total RNA from tissues and cells according to the instructions. The extracted total RNA was measured with NanoDorp. 5 μg of total RNA was used for reverse-transcription into cDNA using miRNA qPCR Quantitation Kit (GenePharma, Shanghai, China) and PrimeScript RT Reagent Kit (Takara, Japan). StepOnePlus™ System (Applied Biosystems, Foster City, CA, USA) was used for qPCR analysis using SYBR Green Master Mix (Takara, Japan). U6 snRNA and GAPDH genes were used as internal references for relative gene expression by the 2–∆∆Ct method. Sequences of all primers were shown in Supplementary Materials and Methods.

### Cell Counting Kit-8 (CCK-8) analysis

48 hours after transfection, cells were seeded in to a 96 -well plate at a density of 1000 cell/well and cultured in a humidified cell culture incubator for 0, 24, 48, 72 and 96 hours, respectively. Subsequently, 10 μL CCK8 reaction solution (Solarbio, Japan) was added to the cell culture at indicated time point and incubated for 1 hour in a humidified cell culture incubator. The light absorption value (OD value) in each condition was captured at 450 nm wavelength.

### Transwell migration and invasion assays

Cell invasion and migration assays were performed using the 8-μm Transwell chamber (Corning, NY, USA) and placed into the 24-well plates. Transwell coated with Matrigel (0.5 mg/mL, BD Biosciences) was used for invasion assay. PCL5 or HepG2 cells were trypsinized and suspended in FBS-free DMEM. 5 × 10^5^ cells were inoculated in the upper Transwell chamber and DMEM with 10% FBS was added in the lower chamber as the chemoattractant. After 24 hours, culture medium was discarded and the cells were fixed with 4% paraformaldehyde at room temperature for 10 mins and stained with 0.5% crystal violet (Sigma, Germany) for 20 mins. Cells were photographed under Leica AM6000 microscope (Leica, Wetzlar, Germany), and the number of invading cells was counted.

### Apoptosis assay

Apoptosis assay was performed using annexin V-FITC and PI kit (Beyotime, China). 48 hours post-transfection, HepG2 and PCL5 cell lines were trypsinized and washed twice with 1 x PBS, and resuspended in the staining solution. 5 μL Annexin V-FITC and 5 μL PI were added to the 1000 μL cell resuspension with 1 million cells and incubated for 30 mins in the dark. Stained cells were washed once with 1xPBS and resuspended in 500 μL staining buffer. The percentage of apoptotic cells was detected by FACScan Flow Cytometer (Biosciences, USA).

### Dual-luciferase reporter assay

To study the functional interaction between microRNA and its targets, fragments contain the wild-type binding site (WT) or the mutated sequence (MUT) were synthesized and cloned into PmirGLO firefly luciferase vector respectively (Promega, CA, USA). Cells were co-transfected with 0.5 µg reporter plasmid and 0.5 µg Renilla luciferase (hRlucneo) control plasmid in the presence of miR-421 mimic or miR-NC in a 96-well plate (5000 cells/well) using Lipofectamine 3000 reagent according to the manufacturer’s instructions. After 48 hours, the relative luciferase activities were determined using Dual-Luciferase Reporter Assay Kit (Promega) on a luminescence microplate reader. The relative firefly luciferase activity in the reporter was normalized to that of Renilla luciferase control plasmid.

## Western blotting analysis

80% confluent cells into a 6-well plate were lysed using the RIPA lysis buffer (Applygen, Beijing) to extract the total protein. Lysed cells were centrifuged at 14,000 rpm for 10 mins and the supernatant containing total protein was quantified by a BCA Protein assay kit (Beyotime, Shanghai, China). 10 ug protein was used for SDS-PAGE electrophoresis and transferred to PVDF membranes (Millipore, Billerica, MA). After blocking with 5% skimmed milk for 1 hour, the membranes were incubated with primary antibodies under 4°C overnight. After wash with TBST buffer, second antibodies conjugated with horseradish peroxidase were added to the membranes for 1-hour incubation. Protein bands were developed using an ECL reagent (Beyotime, China), and Image J software (National Institutes of Health, Bethesda, MA, USA) was utilized for densitometry analysis.

## Data analysis

Data were analyzed by SPSS22.0 (SPSS, Inc., Chicago, IL, USA), and GraphPad Prism 7.0 software (GraphPad, Inc., La Jolla, CA, USA) was utilized for creating graphs. All experiments were repeated three times and measurement data were displayed as mean ± SD. Student’s t-test or one-way ANOVA was adopted for significance analysis. *P* < 0.05 was considered as statistically significant (*P < 0.05; **P < 0.01; ***P < 0.001; ****P < 0.0001).

## Results:

This study analyzed LINC00839 expression level and investigated its functional roles and downstream targets in HCC. We showed that LINC00839 was highly expressed in HCC, and high LINC00839 expression was correlated with a poor prognosis in HCC patients. Through different functional assays, we demonstrated the oncogenic role of LINC00839 in HCC cells. Mechanistically, LINC00839 served as the sponge of miR-144-3p, which relived the suppression on WTAP expression in HCC.

## LINC00839 is upregulated in HCC tissues and cells

We first used StarBase v3.0 (http://starbase.sysu.edu.cn/) to comparing LINC00839 expressions in 374 HCC cancer and 50 normal liver tissues. LINC00839 level of HCC samples was significantly upregulated as compared to normal samples (Figure. 1a). Similarly, qRT-PCR analysis revealed that LINC00839 expression in 60 HCC samples collected in this study was significantly elevated as compared to the paired normal tissues (Figure. 1b). We also detected the significant upregulation of LINC00839 expression in HCC cell lines (Huh6, Huh7, SK-hep1, HepG2, and PLC5) when compared to that of normal hepatocyte cell line (LO2) (Figure 1c). To assess whether LINC00839 expression was associated with patient survival, the overall survival (OS) of HCC cases from KM-plotter (https://kmplot.com/analysis/index.php?p=service&cancer=pancancer_rnaseq) (Figure. 1d left) as well as OS of our patients (Figure. 1d right) were analyzed based on LINC00839 expression. HCC cases with low LINC00839 expression generally showed better OS.

Taking the median expression value of LINC00839 in HCC samples in Figure 1a as a threshold, 60 HCC cases were classified into low-expression group (n = 30) and high-expression group (n = 30). The association of LINC00839 expression with clinicopathological data of HCC was analyzed using Chi-square test (Table 1). High LINC00839 expression level was significantly associated with tumor size, lymph node metastasis (LNM) and a poor tumor differentiation. Together, this data suggest that elevated LINC00839 expression may facilitate HCC progression.

**Table 1.** Correlation of linc00839 expression with clinicopathologic features of  hepatocellular carcinoma

	LINC00839		
Patients	High (n = 30)	Low (n = 30)	*P* value
**Age (years)**			0.1965
<58	17	12	
≥58	13	18	
**Gender**			0.4475
Male	27	25	
Female	3	5	
**HBV infection**			0.176
Positive	22	17	
Negative	8	13	
**Liver cirrhosis history**			0.3006
Yes	18	14	
No	12	16	
**Tumor size**			0.0201
≤5 cm	11	20	
>5 cm	19	10	
**AFP**			0.152
≤200	6	11	
>200	24	19	
**Lymph node metastasis**			0.0149
No	15	24	
Yes	15	6	
**Clinical satge**			0.0195
I+ II	9	18	
III+IV	21	12	
**Tumor differentiation**			0.0384
Well+moderate	12	20	
Poor	18	10	

## Silencing LINC00839 inhibits the proliferation, invasion, migration and promotes apoptosis in HCC cells

We next synthesized two siRNAs for knocking down LINC00839 (si-LINC00839^1#^ and si-LINC00839^2#^). qRT-PCR results showed that LINC00839 level in HepG2, and PLC5 cell lines was significantly reduced after transfected with siRNAs (Figure 2a). Si-LINC00839^1#^ shoed a higher knockdown efficiency and was chosen for subsequent experiments. CCK-8 assays showed that LINC00839 knockdown significantly suppressed cell proliferation in both cell lines (Figure 2b). Colony formation experiments revealed that silencing LINC00839 impaired colony formation ability of HepG2 and PLC5 cells (Figure 2c). In addition, Transwell assays demonstrated the impaired cell invasion and migration ability after LINC00839 silencing (Figure 2d,e). Apoptosis assay by flow cytometry further revealed an increased percentage of apoptotic cells in HepG2 and PLC5 cells after LINC00839 knockdown (Figure 2f).

## LINC00839 serves as a sponge for miR-144-3p

We next sought to search for the downstream target of LINC00839. The possible interaction microRNAs of LINC00839 were predicted through the online bioinformatics analysis tool DINAN and miRcode. We found 13 targets miRNAs shared by the two databases (sFig4). We selected three miRNAs with top prediction scores including miR-338-3p (score: 1.000), miR-144-3p (score: 0.995) and miR-20b-5p (score: 0.976). We then evaluated the expression level of these three miRNA candidates after LINC00839 knockdown by qR-PCR in HepG2 cells. Only miR-144-3p was significantly upregulated after LINC00839 knockdown (sFig5). Therefore, we reasoned that miR-144-3p was a potential target regulated by LINC00839 (Figure 3a).

In both HepG2 and PLC5 cells, miR-144-3p level increased after si-LINC00839 transfection (Figure 3b). We next performed dual-luciferase assay to confirm the functional interaction. The results showed that the reporter containing wild-type binding site (Luci-LINC00839-WT) was significantly inhibited by miR-144-3p mimic, while miR-144-3p mimic showed no effect on mutated reporter (Luci-LINC00839-mut) or empty reporter (Luci-NC) (Figure 3c).

Moreover, we also found that LINC00839 overexpression significantly downregulated the expression levels of miR-144-3p (sFig6A & 6B). Notably, the expression level of LINC00839 were unchanged after transfected with miR-144-3p inhibitor (sFig6C & 6D) or miR-144-3p mimic (sFig6E & 6 F). These results indicate that LINC00839 sponges miR-144-3p to negatively regulate its expression, while miR-144-3p does not affect LINC00839 level. Consistently, miR-144-3p level was markedly decreased in HCC samples from the database (Figure 3d) and in the 60 HCC samples collected in this study (Figure 3e). Furthermore, we conducted a Spearman correlation analysis and found that LINC00839 expression level was negatively correlated with the level of miR-144-3p levels in 60 HCC samples (Figure 3f). Overall, our data indicate that LINC00839 serves as a sponge for miR-144-3p.

## miR-144-3p mediates the biological functions of LINC00839 in HCC cells

**Figure 4.**
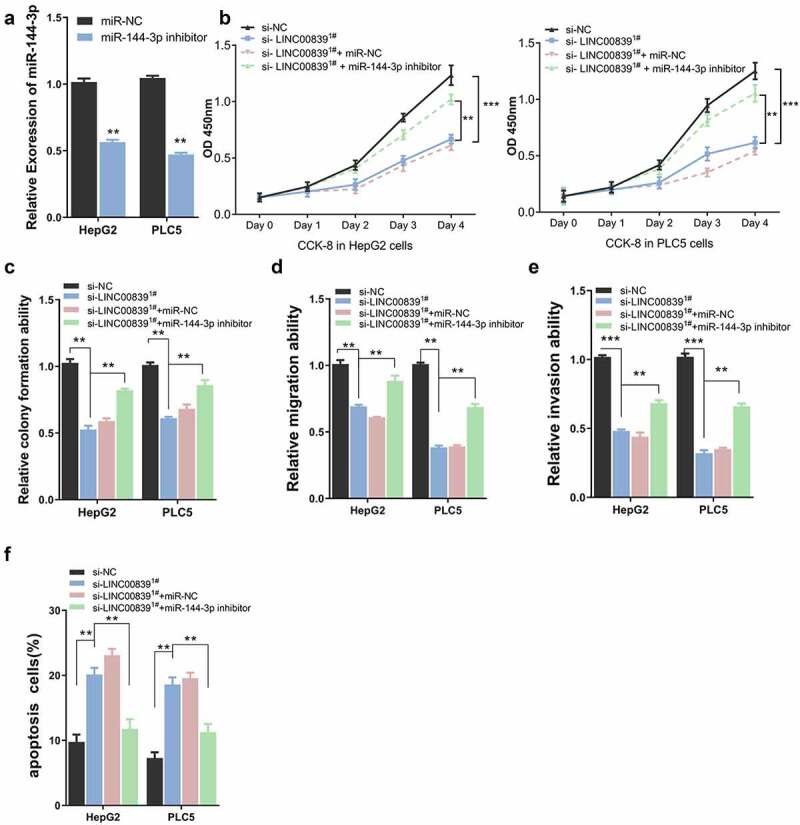
The effect of miR0144-3p on cell phenotypes. a) miR-144-3p knockdown efficiency of miR-144-3p inhibitor. b) miR-144-3p inhibitor partially rescued the inhibition effect on cell proliferation of si-LINC00839 in HCC cells. c) miR-144-3p inhibitor partially recovered colony formation ability after LINC00839 silencing. d-e) miR-144-3p inhibitor partially rescued the inhibition effect of si-LINC00839 on migration and invasion ability. f) miR-144-3p inhibitor prevented the apoptotic effect of LINC00839 silencing. The data were shown as mean ± SD of three independent experiments. **, P < 0.01; ***, P < 0.001

We next performed rescue experiment to assess whether inhibiting miR-144-3p affect the effect of LINC00839 silencing. Cells were transfected with si-NC, si-LINC00839, si-LINC00839+ miR-NC or si-LINC00839+ miR-144-3p inhibitor. miR-144-3p inhibitor could significantly downregulate miR-144-3p level in HepG2 and PLC5 cells (Figure 4a). CCK-8 assays revealed that miR-144-3p knockdown attenuated the proliferation suppression by LINC00839 knockdown (Figure 4b). Similar results were observed in clone formation experiments: si-LINC00839 significantly reduced cell colony formation, while miR-144-3p inhibitor co-transfection partially restore the colony formation capacity (Figure 4c). The application of miR-144-3p inhibitor also restored the cell migration and invasion ability after LINC00839 silencing ((Figure 4d,e), while the apoptosis induction by si-LINC00839 was abrogated (Figure 4f). Therefore, miR-144-3p is involved in the mediation of biological functions of LINC00839 in HCC cells

## miR-144-3p regulates WTAP expression in HCC cells

**Figure 5.**
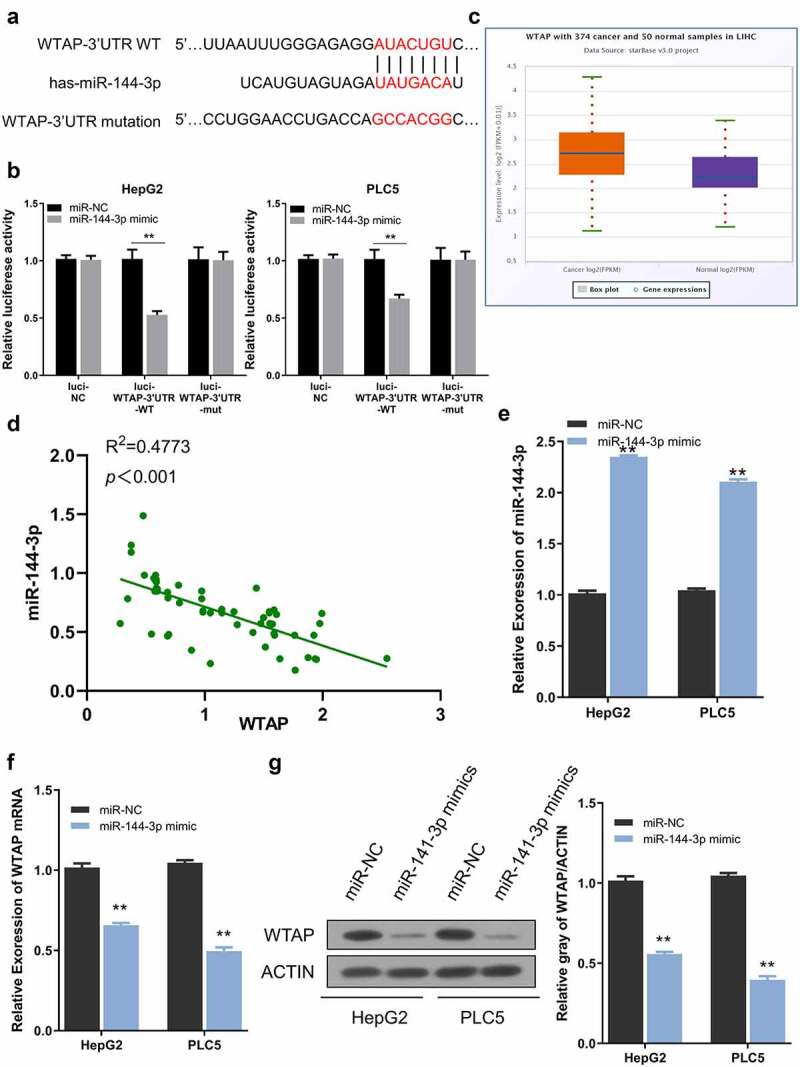
WTAP is a downstream target of miR-144-3p. a) The predicted binding sites between WTAP and miR-144-3p. b) Dual-luciferase assay using reporter with wild-type binding site (Luci-WTAP-WT), mutated reporter (Luci-WTAP-mut) or empty reporter (Luci-NC) in the presence of MiR-144-3p mimic or miR-NC. c) The expression level of WTAP in HCC tissues from the database. d) A negative correlation between WTAP and miR-144-3p expression in 60 HCC samples. e) miR-144-3p mimic upregulated miR-144-3p level. f) miR-144-3p mimic downregulated WTAP mRNA level by qRT-PCR. g) Mi-144-3p mimic downregulated the protein level of WTAP. The data were shown as mean ± SD of three independent experiments. **, P < 0.01; ***, P < 0.001

As predicted by the TargetScan website, a miR-144-3p binding site was detected within the WTAP 3ʹ-UTR noncoding region, and the sequence was shown in Figure 5a. To confirm this functional interaction, PLC5 and HepG2 cells were subjected to dual luciferase reporter assay. The results showed that the reporter containing wild-type binding site (Luci-WTAP-3ʹUTR-WT) was significantly inhibited by miR-144-3p mimic, while miR-144-3p mimic showed no effect on mutated reporter (Luci-WTAP-3ʹUTR-mut) or empty reporter (Luci-NC) (Figure 5b). WTAP mRNA expression was markedly increased in HCC cases from the database (Figure5c). In 60 HCC samples collected in this study, WTAP expression showed a significant negative correlation with miR-144-3p level (Figure5d). Besides, after miR-144-3p mimic transfection which significantly elevated miR-144-3p level (Figure 5e), both the mRNA and protein levels of WTAP were reduced after miR-144-3p overexpression (Figure 5f,g). Collectively, these results indicate that miR-144-3p negatively targets WTAP expression in HCC cells.

## LINC00839 enhanced HCC malignant behaviors by modulating miR-144-3p/WTAP axis

**Figure 6.**
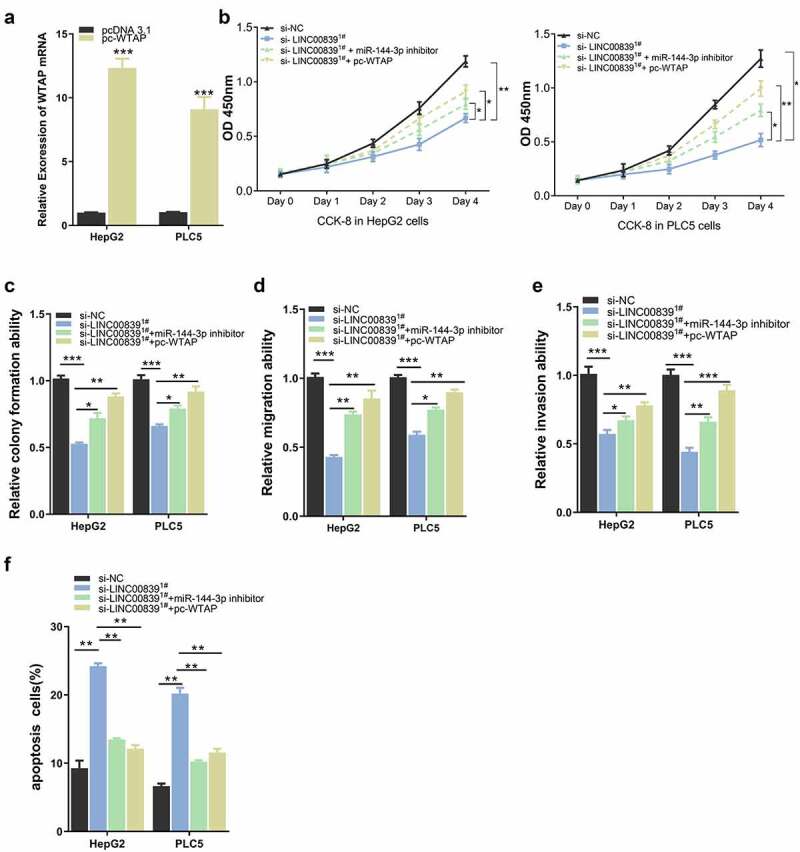
The effect of WTAP overexpression on cell phenotypes. a) The overexpression efficiency after transfecting with pc-WTAP expression plasmid. b) WTAP overexpression rescued the inhibition effect of si-LINC00839 on cell proliferation. c) WTAP overexpression enhanced cell colony formation after LINC00839 silencing. d-e) WTAP overexpression rescued the inhibition effect of si-LINC00839 on the migration and invasion ability of HCC cells. f) WTAP overexpression prevented cell apoptosis induced LINC00839 silencing. The data were shown as the mean ± SD of three independent experiments. *, P < 0.05; **, P < 0.01; ***, P < 0.001

We next sought to further explore whether miR-144-3p/WTAP axis mediates LINC00839 roles in HCC cells. pc-WTAP expression vector was constructed and transfected into cells to overexpress WTAP (Figure 6a). CCK8 assays revealed that both miR-144-3p inhibitor or pc-WTAP rescued the inhibitory effect of si-LINC00839 on cell proliferation (Figure 6b), and similar effect were observed in colony formation assay (Figure 6c). The co-transfection of miR-144-3p inhibitor or pc-WTAP also restored the cell migration and invasion ability after LINC00839 silencing (Figure 6d, 6E), while the apoptosis induction by si-LINC00839 was abrogated (Figure 6f). Together, these data demonstrated that miR-144-3p/ WTAP axis serves as downstream regulators of LINC00839.

## Discussion

LncRNAs have gradually become a research hotspot in tumors, which are implicated in cancer genesis and progression [2]. LINC00839 has been studied in osteosarcoma and breast cancer, in which it is upregulated and functions as an oncogenic factor [8,9], but the role of LINC00839 has not been reported in HCC. our study revealed that LINC00839 is highly expressed in HCC cells and tissues. LINC00839 plays a carcinogenic effect in HCC, and its silencing impairs the malignant phenotype of HCC cells. We further demonstrated that miR-144-3p/ WTAP axis serves as downstream regulators of downstream regulators of LINC00839 in HCC cells.

As reported by several studies, miR-144-3p serves as a tumor suppressor [10,23]. Consistently, our study showed that miR-144-3p is downregulated in HCC and functions as a tumor suppress gene [17,24,25]. Based on our data, miR-144-3p overexpression attenuated the inhibitory effect of LINC00839 silencing, and it shows an negative correlation with LINC00839 expression in HCC samples. These data indicate that LINC00839 negatively regulates miR-144-3p expression to exert oncogenic functions.

Substantial evidence shows that abnormal RNA posttranscription modification is implicated in the progression of HCC [26]. RNA methylation, a type of reversible post-transcriptional modification, has attracted increasing attention since its dysregulation is associated with HCC progression [20,27,28]. WTAP functions as a writer in m6A modification, which stabilizes the activities of METTL3 and METTL14 to facilitate m6A modification. WTAP dysregulation is related to different kinds of tumors, such as cholangiocarcinoma, renal cell carcinoma, colon cancer, and acute myeloid leukemia [29–32]. It also has been demonstrated that WTAP facilitates the progression of HCC [22]. Taken together, the available evidence suggests that WTAP could serve as an oncogene. Consistent with this notion, we showed that WTAP overexpression could enhance the malignant phenotype of cells, and it is directly regulated by miR-144-3p in HCC.

Collectively, we demonstrated the oncogenic role of LINC00839 in HCC and revealed the interplay of LINC00839/miR-144-3p/WTAP axis in regulating the malignant phenotype of CC cells. However, since LINC00839 may have multiple downstream miRNA target, a holistic regulatory network by LINC00839 remains to be studied. Other downstream targets regulated by LINC00839 may also play functional roles in tumorigenesis, such as miR-144-3p and Lin28B [8,9]. It is also worth noting that our assays were carried out in cultured cells. The role of LINC00839/miR-144-3p/WTAP axis in tumorigenesis need to be validated in animal model. Furthermore, antisense oligonucleotide targeting LINC00839 should be developed to evaluate the therapeutic value of targeting LINC00839 *in vivo*.

## Conclusion

In conclusion, our results illustrated the oncogenic effect of LINC00839 in HCC, which is indispensable for the malignant behaviors of HCC cells. LINC00839 acts as a miR-144-3p sponge, and LINC00839 upregulation maintain WTAP expression by negatively regulating miR-144-3p. Collectively, our data suggest that LINC00839 might serve as a potential therapeutic target and prognostic marker for HCC.

## Supplementary Material

Supplemental Material

## Highlights:

- LINC00839 is upregulated with HCC cells and tissues.
- LINC00839 knockdown impairs the proliferation, invasion, migration of HCC cells.
- miR-144-3p and WTAP regulate the proliferation, invasion, migration of HCC cells.
- LINC00839 promotes malignant phenotype of HCC via miR-144-3p/WTAP axis.
