# Supplementary figures and images for: LINC00839/miR-144-3p/WTAP (WT1 Associated protein) axis is involved in regulating hepatocellular carcinoma progression
Source: Bioengineered. 2021 Nov 30;12(2):10849–61. doi: 10.1080/21655979.2021.1990578 (PMC8809969; doi:10.1080/21655979.2021.1990578)

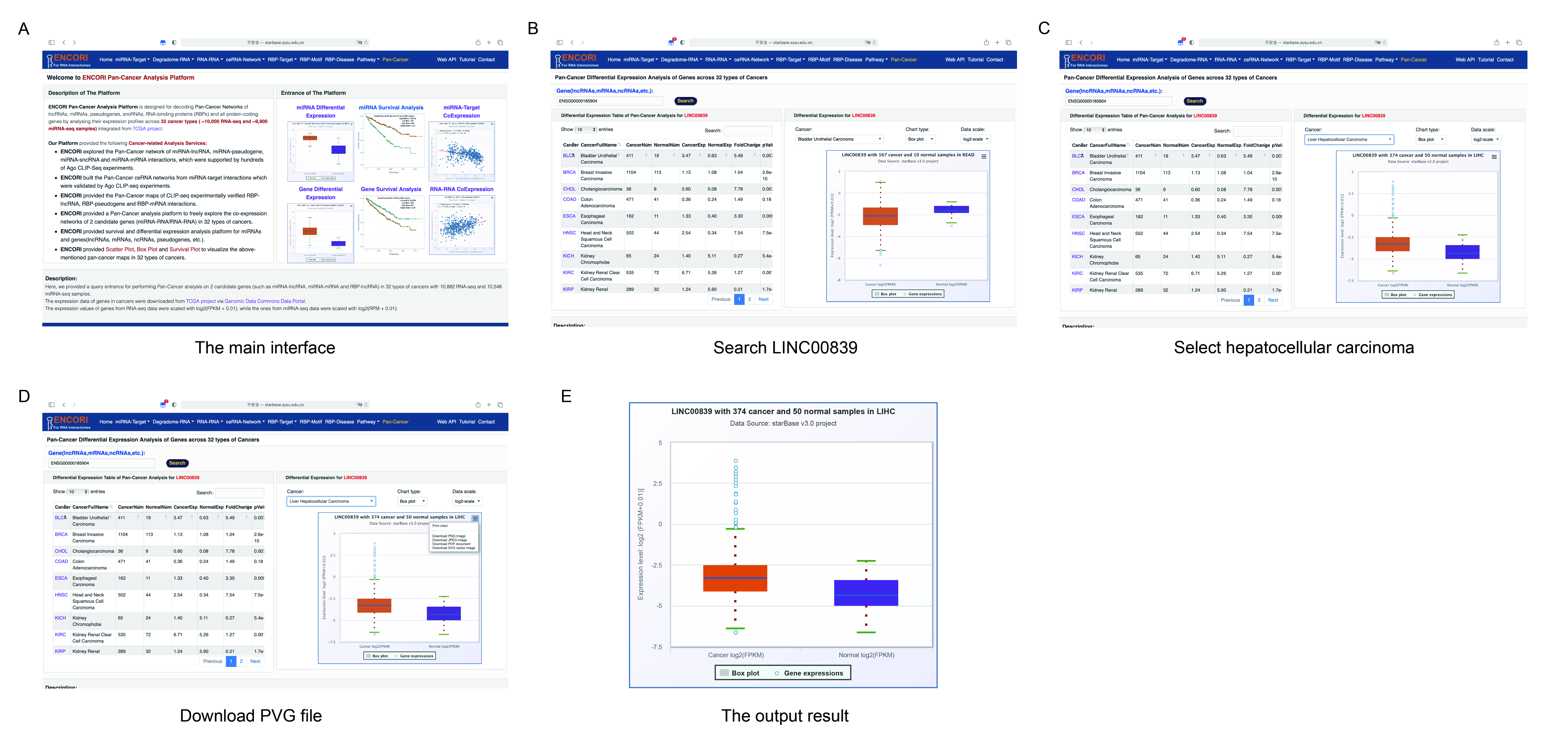

Supplement: Supplemental Material [file KBIE_A_1990578_SM0083.zip › sFigure 1.tif]

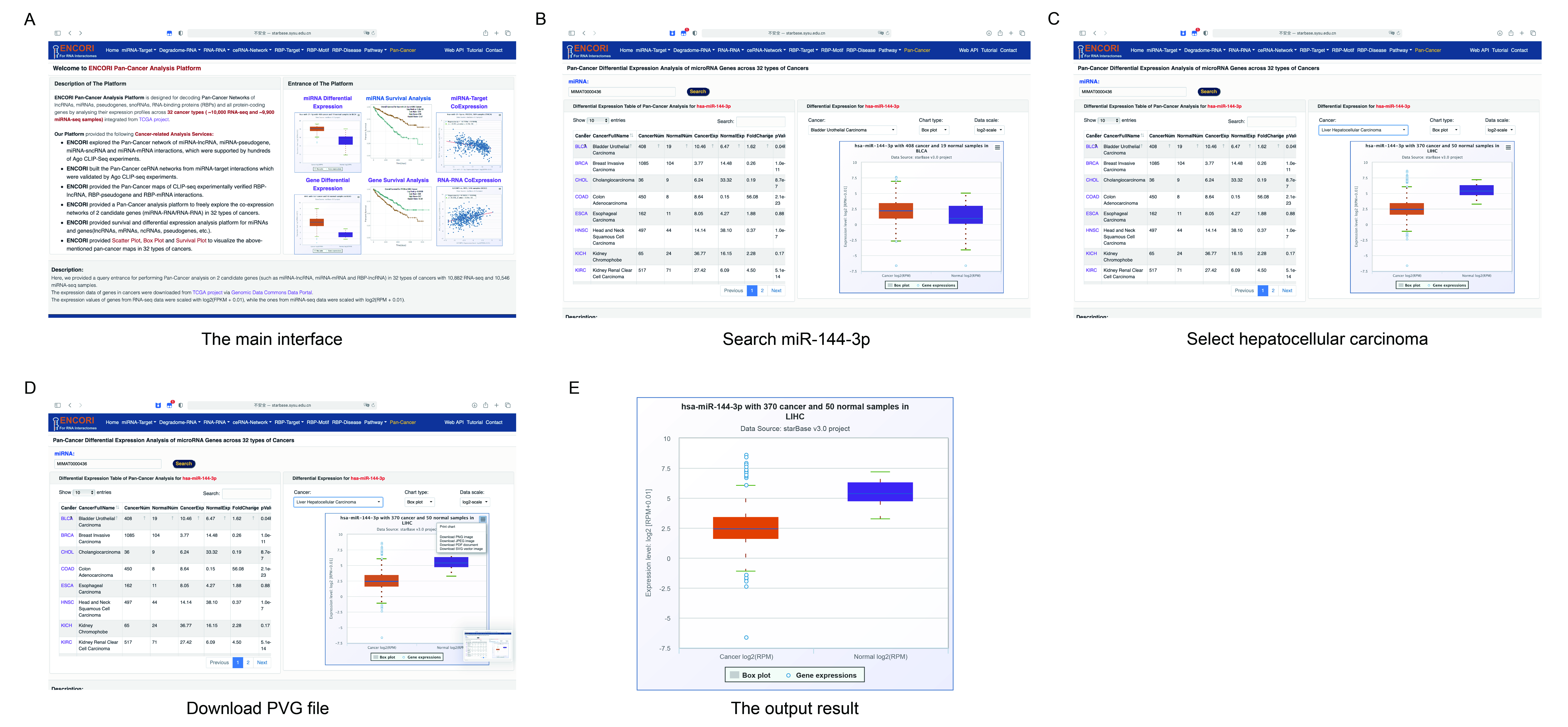

Supplement: Supplemental Material [file KBIE_A_1990578_SM0083.zip › sFigure 2.tif]

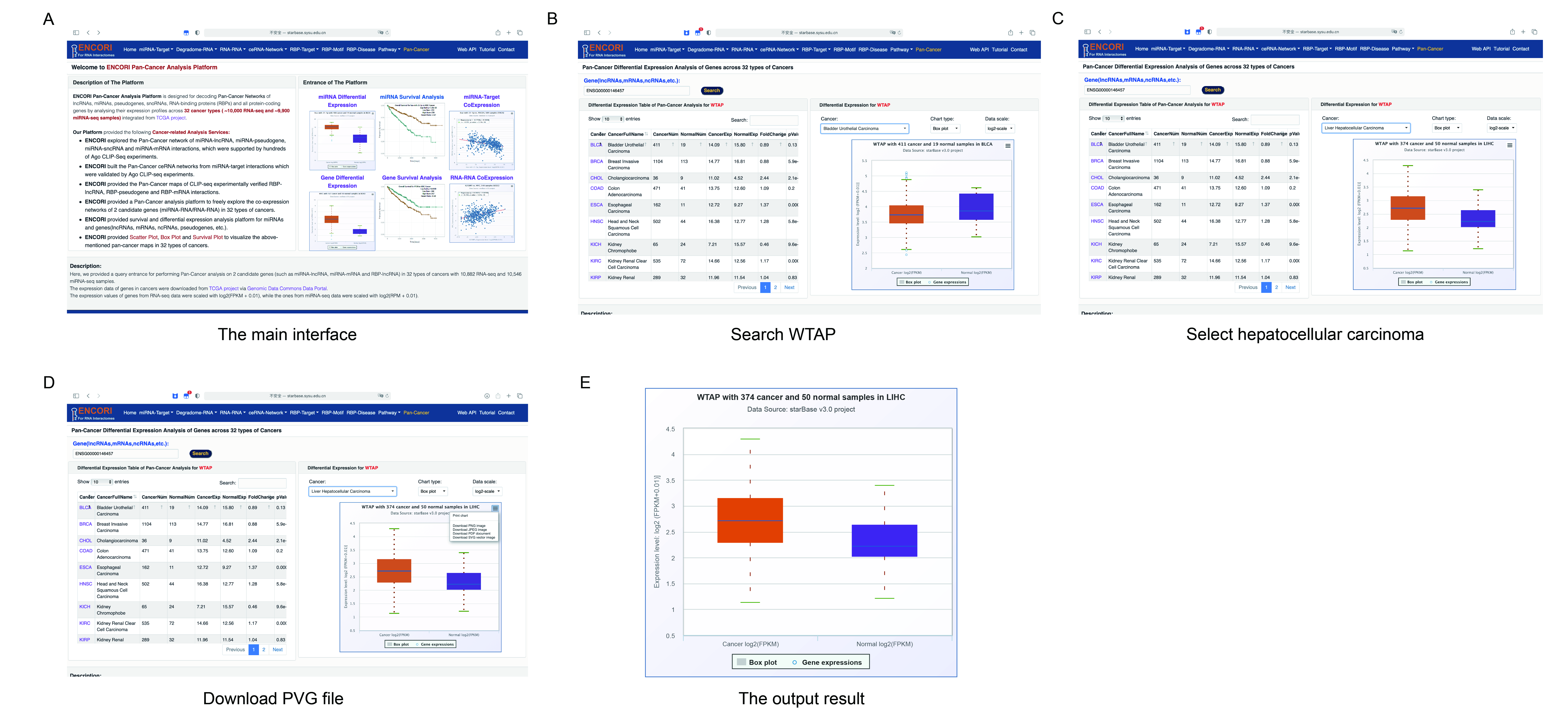

Supplement: Supplemental Material [file KBIE_A_1990578_SM0083.zip › sFigure 3.tif]

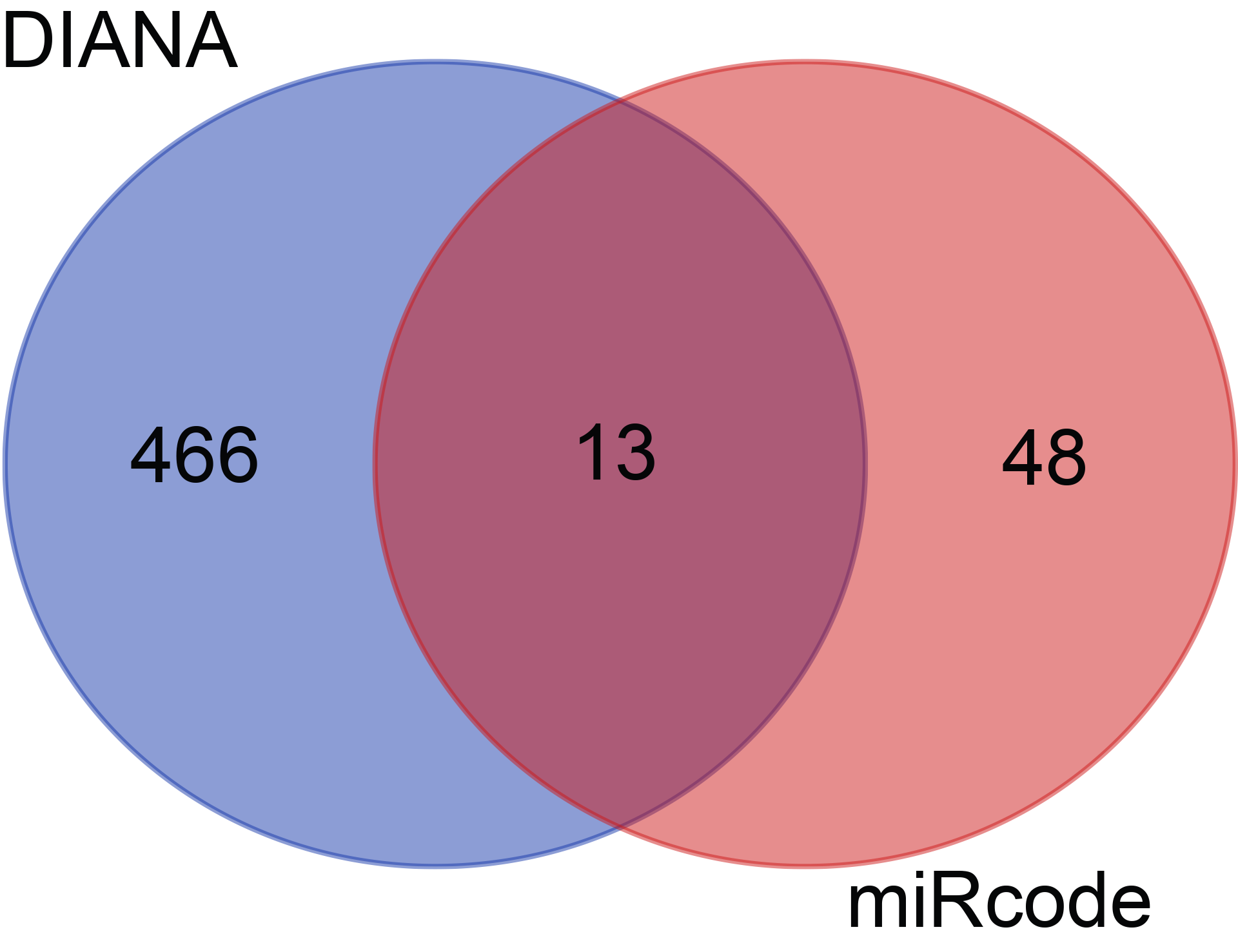

Supplement: Supplemental Material [file KBIE_A_1990578_SM0083.zip › sFigure 4.tif]

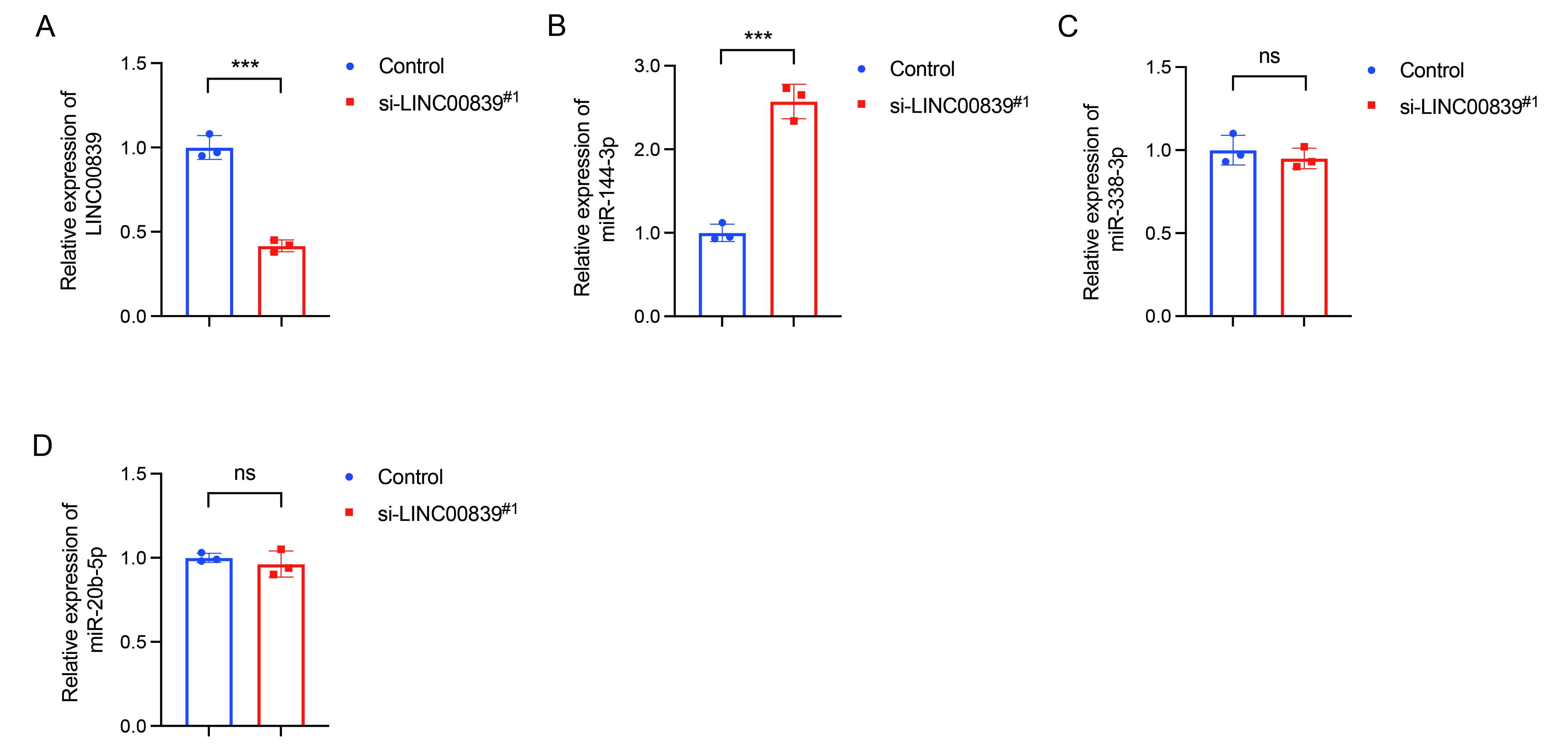

Supplement: Supplemental Material [file KBIE_A_1990578_SM0083.zip › sFigure 5.tif]

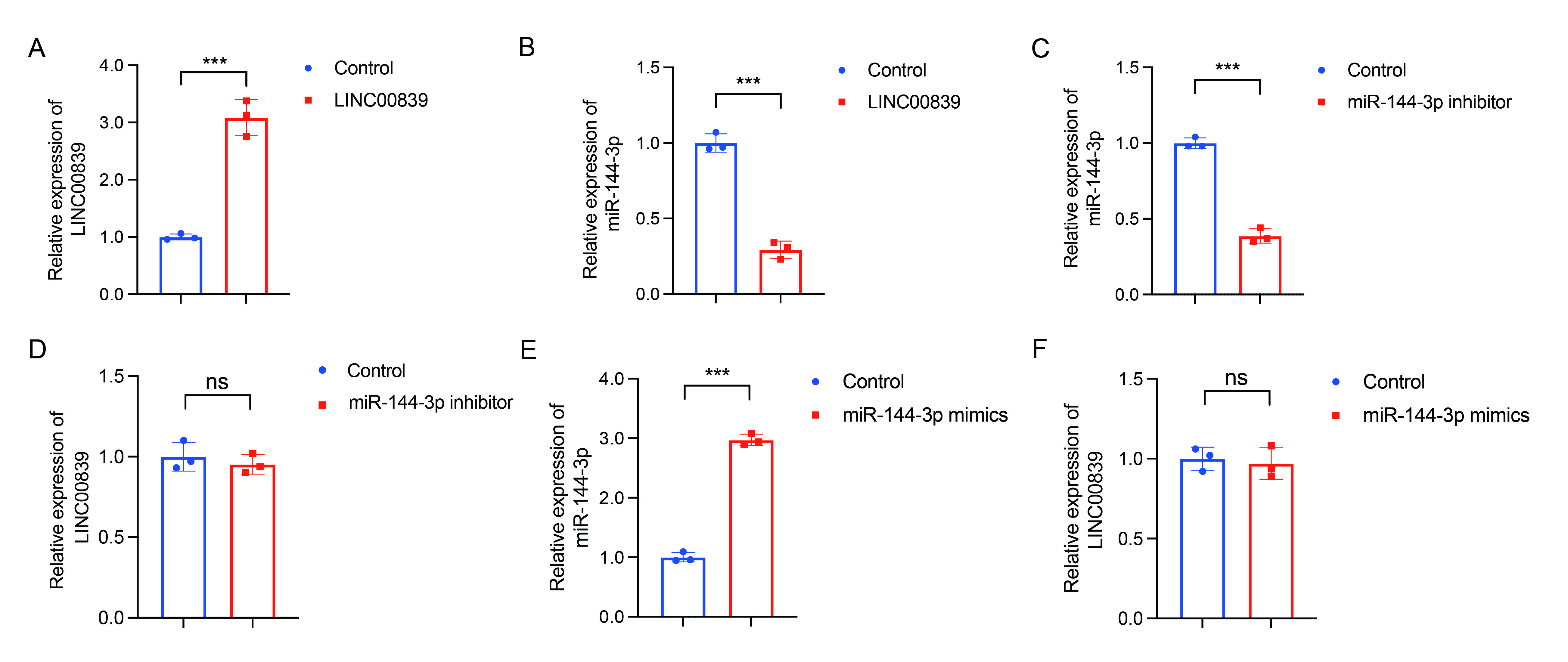

Supplement: Supplemental Material [file KBIE_A_1990578_SM0083.zip › sFigure 6.tif]
